# Tracing tumor-neuronal connectivity in glioblastoma

**DOI:** 10.1093/neuonc/noaf005

**Published:** 2025-01-11

**Authors:** Sean E Lawler

**Affiliations:** Department of Pathology and Laboratory Medicine, Legorreta Cancer Center, Brown University, Providence RI 02903, USA

Over recent years, our increased appreciation of the importance of tumor-neuronal synaptic connectivity in glioblastoma and other brain cancers has changed the way we think about these tumors from both biological and therapeutic perspectives.^1–3^ Studies in mouse models and human brain tumors have clearly demonstrated the existence of synaptic interactions between tumor cells and normal neurons in brain cancers and have supported their role in driving tumor growth and promoting resistance to therapy. However, we still do not fully understand the extent of these networks, the nature of the signals involved, and how to target them effectively.

In a recent study published in *Cell*, the authors employed retrograde tracing to investigate neuron-tumor networks.^4^ Retrograde tracing is a well-established technique used in neuroscience to visualize cells connected to one another via neuronal synapses. Here, the authors used a modified rabies virus that can only spread trans-synaptically to neighboring neurons, allowing the efficient tracing of neurons that directly synapse to glioblastoma cells that had been pre-infected with the virus. Patient-derived glioblastoma cells engineered to express the fluorescent protein mCherry were infected with rabies virus encoding green fluorescent protein (GFP) and co-cultured with human neurons and organotypic human brain slices, or implanted into the brains of recipient mice as patient-derived xenograft (PDX) models. GFP expression in co-cultured neurons was identified in each of the model systems, indicating direct functional synaptic connections with glioblastoma cells. Longitudinal imaging, electrophysiology, functional assays, and molecular characterization were all employed to examine this in detail. The study revealed that neuronal connections emerge rapidly when patient-derived glioblastoma cells are grown in the presence of neurons and that these connections can be far reaching. Tumor cells can establish transient connections with neurons independently of their type, and neuronal activity increases the tumor connectivity. Greater connectivity was observed in the more invasive tumor models. The study also showed that neurons connected with tumor cells behave in a normal manner and retain their integration with other neurons that are not connected to tumor cells. Interestingly, connected neurons can become hyperexcitable when treated with irradiation consistent with clinical observations of seizures in patients. Disruption of these connections sensitized tumor cells to irradiation, supporting development of this approach in the clinical setting.

In each of the model systems, neuronal connections were established quickly (within hours) and resulted in post-synaptic currents indicating their biological integration. Characterization of connected neurons showed that they remained viable and retained their normal electrical activity. The observation that neurons retain their normal functions was supported by longitudinal multiphoton microscopy studies that showed similar dendritic spine behavior across models and between connected and unconnected neurons, at least at the earlier stages of interactions. These data suggest that tumor-connected neurons maintain their function and integration into broader circuits.

When comparing different tumor models (14 patient-derived models were used in the study), the authors identified a correlation between the invasive behavior of each model and “synaptogenic scores” (based on synaptic gene expression signatures). Tumor models with increased invasion also showed greater neuronal connectivity and there was a correlation with a recently described epigenetic neural signature and invasion/connectivity,^5^ which also was linked to poorer survival. Thus, synaptogenic potential, invasion, and tumor-neuron connectivity appear to be important features and are correlated in glioblastoma.

In mouse PDX models, long-range projections were observed throughout the brain including the contralateral hemisphere. As may be expected, the patterns of connectivity were dependent on tumor location, with more dispersed connectivity in cortical compared with striatal tumors, which increased over time. In these mouse models, optogenetic stimulation of glutamatergic neurons increased invasion, consistent with previous studies.^1–3^

Data showed that glioblastoma cells can connect to a range of neuronal subpopulations, including glutamatergic, and cholinergic excitatory neurons, as well as GABAergic inhibitory neurons. Detailed examination of cholinergic signaling showed that the acetylcholine receptor (CHRM3) was highly expressed in glioblastoma cells, and its antagonist atropine blocked signaling events. CHRM3 knockdown led to impaired cortical tumor growth, suggesting acetylcholine is a key mediator in neuron-tumor communication.

From the perspective of standard of care, the authors found that radiotherapy reduced the number of glioblastoma cells after treatment, but that the number of connected cells were higher. This suggests a selection for connected glioblastoma cells, and a potential resistance mechanism as has been suggested in previous studies.^1–3^ An increase in action potential bursting activity (neuronal hyperexcitability) was observed following radiotherapy. This is consistent with observations of increased seizures in some glioblastoma patients following radiotherapy. Inhibition of AMPA receptors with the drug perampanel disrupted connectivity and reduced tumor progression. Finally, the authors used an engineered rabies virus to ablate tumor-connected neurons, which showed a significant reduction in tumor cells.

These data provide a new platform for the study of neuron–glioblastoma interactions in multiple types of co-culture model. Furthermore, these models may be particularly useful in future studies to identify approaches that could block this connectivity therapeutically. The therapeutic relevance of this approach was illustrated in the studies showing that irradiation led to hyperexcitability. Furthermore, this platform could be applicable to other brain tumor types in similar ways.

We still do not understand the long-term effects of neuron-tumor network formation, as this study focuses on the initial and early stages of interactions. This is much more challenging using these types of models, which are short-term in their nature. Another limitation is that rabies may not label all connected neurons and rabies can be neurotoxic over time, which also could limit long-term studies. Despite the interest generated by this data, we are still awaiting the clinical application of these approaches. Targeting tumor–neuron interactions would potentially slow progression, reduce invasion, and sensitize tumors to chemoradiation. The study also shows that there is a spectrum of interactions between distinct neuronal subtypes and glioblastoma cells, and also that there is heterogeneity between patients and due to tumor location. Understanding this complexity may be of importance in the application of broadly effective or personalized therapies.

These data strongly support the development of combination therapies that could interfere with tumor-neuronal interactions alongside standard of care and overcome resistance driven by neuron-tumor connectivity. Moreover, this type of tracing approach could be applied more widely to understand responses of glioblastoma to therapeutics, and to elucidate the mechanisms involved, allowing the development of innovative and effective new approaches.

## References

[1] Winkler F, Venkatesh HS, Amit M, et al Cancer neuroscience: state of the field, emerging directions. Cell. 2023;186(8):1689–1707. 37059069 10.1016/j.cell.2023.02.002 PMC10107403

[2] Venkatesh HS, Tam LT, Woo PJ, et al Targeting neuronal activity-regulated neuroligin-3 dependency in high-grade glioma. Nature. 2017;549(7673):533–537. 28959975 10.1038/nature24014 PMC5891832

[3] Venkataramani V, Tanev DI, Strahle C, et al Glutamatergic synaptic input to glioma cells drives brain tumour progression. Nature. 2019;573(7775):532–538. 31534219 10.1038/s41586-019-1564-x

[4] Tetzlaff SK, Reyhan E, Layer N, et al Characterizing and targeting glioblastoma neuron-tumor networks with retrograde tracing. Cell. 2024;S0092-8674(24)01276-5. 10.1016/j.cell.2024.11.002 39644898

[5] Drexler R, Khatri R, Sauvigny T, et al A prognostic neural epigenetic signature in high-grade glioma. Nat Med. 2024;30(6):1622–1635. 38760585 10.1038/s41591-024-02969-w PMC11186787

